# Characteristics of central cortex and upper-limb flexors synchrony oxygenation during grasping in people with stroke: a controlled trial study protocol

**DOI:** 10.3389/fnhum.2024.1409148

**Published:** 2024-08-29

**Authors:** Jiang-Li Zhao, Pei-Ming Chen, Tao Zhang, Hao Xie, Wen-Wu Xiao, Shamay S. M Ng, Chu-Huai Wang

**Affiliations:** ^1^Department of Rehabilitation Medicine, The First Affiliated Hospital, Sun Yat-sen University, Guangzhou, Guangdong, China; ^2^Department of Rehabilitation Sciences, The Hong Kong Polytechnic University, Hong Kong SAR, China

**Keywords:** rehabilitation, stroke, motor function, functional near-infrared spectroscopy, oxygenation, virtual reality training

## Abstract

**Background:**

Upper limb motor impairment is a common consequence of stroke, and the effectiveness and underlying mechanisms of rehabilitation therapy for improving upper limb function remain uncertain. Functional near-infrared spectroscopy, a reliable wearable neuroimaging technique, holds promise for investigating brain activity during functional tasks. This study aims to explore the synchronous oxygenation characteristics of the central cortex and upper-limb flexors during a grasping task and investigate the rehabilitation mechanisms of upper limb motor function in individuals with stroke.

**Methods:**

Participants with stroke who demonstrate the ability to grasp and lift cubic wood blocks of different sizes (2.5cm^3^, 5cm^3^, and 10cm^3^) using their affected hand will be divided into three groups: A, B, and C. Each group will consist of twenty stroke patients, resulting in a total of sixty participants with stroke. Additionally, twenty matched healthy subjects will be enrolled as a control group. Comprehensive assessments will be conducted before and after the intervention, including blood oxygen parameter monitoring of the cerebral cortex and upper limb flexors using fNIRS during the grasping task. Other assessments will include MyotonPRO, the Modified Ashworth Scale, the upper extremity section of the Fugl–Meyer Assessment, the Action Research Arm Test, and the Modified Barthel Index. The study will be undertaken between January 2024 and September 2025.

**Conclusions:**

The results of this trial will provide an in-depth understanding of the Characteristics of central cortex and upper-limb flexors synchronous oxygenation during grasping task and how it may relate to the rehabilitation mechanism of upper limb motor function in people with stroke.

**Clinical trial registration:**

https://www.chictr.org.cn, identifier ChiCTR2400080619.

## Introduction

Stroke, a leading cause of death and disability (GBD Stroke Collaborators, 2019; Stinear et al., 2020), poses a growing challenge due to the aging population and improved survival rates (Li, 2020). Studies have indicated that a significant proportion of stroke patients experience upper limb motor impairment, both in the acute and chronic stages of recovery (Grattan et al., 2019). This impairment directly affects patients’ quality of life and places a substantial economic burden on families and society.

Functional rehabilitation after stroke is believed to result from reorganization within the central nervous system. Understanding the relationship between these neurobiological changes and the recovery process is crucial for helping effective treatment approaches. Non-invasive techniques such as functional magnetic resonance imaging (fMRI), transcranial magnetic stimulation (TMS), and electroencephalography (EEG) have provided valuable insights into the functioning of the human brain (Ward, 2004). Previously, fMRI was employed to investigate intraregional activation and interregional connectivity during bilateral (antiphase and in-phase) and unilateral upper limb movements (Lin et al., 2017), while EEG was utilized to study cognitive anticipatory processes during palm grasping in subacute patients following stroke (Chen et al., 2018). However, these techniques have limitations, such as environmental requirements and human activity, which somewhat hinder the study of cortical activity during functional tasks. In this regard, near-infrared spectroscopy (NIRS) emerges as a novel and non-invasive functional neuroimaging technology. By indirectly assessing cerebral cortex and muscle tissue activity through the detection of blood oxygen parameters [Oxygenated Hemoglobin (HbO), Deoxygenated Hemoglobin (HbR), and Total Oxygenated Hemoglobin (HbT)] based on the neurovascular coupling mechanism, NIRS offers a promising method for investigating neurovascular activity during functional motor tasks. Unlike other techniques, NIRS imposes fewer physical and environmental restrictions and has minimal contraindications (Scholkmann et al., 2014), thereby facilitating the study of cortical activity characteristics in human functional activities.

Current research (Kasahara et al., 2011; Arnemann et al., 2015) in patients following stroke has focus on understanding how focal brain injuries affect brain networks and whether these changes are associated with functional recovery. However, the underlying mechanisms of functional recovery after stroke are still actively being investigated (Ward, 2004). Furthermore, there remains a notable discrepancy between clinical evaluation results and real-life functional abilities of patients (Greeley et al., 2021). To bridge this gap, there is a growing interest in portable neuroimaging techniques that can objectively measure the neurophysiology related to functional behavior (Greeley et al., 2021). Functional near infrared spectroscopy (fNIRS) is a validated and reliable neuroimaging technique that can be used as a wearable device during functional activities (Plichta et al., 2006). It has demonstrated high consistency with fMRI results in detecting cortical activities (Huppert et al., 2017). Some studies have utilized fNIRS to investigate sensorimotor stimulation of the affected hand in patients following stroke though median nerve electrical stimulation (Huo et al., 2019a). However, to date, there have been no reports on the neurophysiological activity of cerebral cortex function region detected by fNIRS during upper limb grasping function in patients following stroke.

While muscle NIRS technology has been widely used in laboratory and exercise settings to observe changes in muscle metabolism and oxygenation (Perrey and Ferrari, 2018), its application has primarily focus on athletes (Giovanelli et al., 2020), healthy people (Beever et al., 2020), and conditions such as cystic fibrosis (Werkman et al., 2016). There is currently a lack of studies utilizing NIRS to examine upper limb muscle hemodynamics in stroke patients, particularly during grasping tasks. Therefore, there is a need to investigate the immediate muscle hemodynamics of upper limb grasping tasks in stroke patients using NIRS, as this will provide valuable insights into the neurophysiological aspects of motor function recovery in this population.

Virtual reality (VR) training which can induce the active participation of patients and provide more repetitive training (Levin et al., 2015), has gained attention as a rehabilitation intervention for improving upper limb function and quality of life in patients following stroke (Lo et al., 2017a). While some studies have shown beneficial effects of VR training on upper limb motor function recovery, a multi-center randomized controlled trial using a commercial gaming system failed to demonstrate superiority over traditional occupational therapy (Saposnik et al., 2016). However, literature analysis suggests that VR systems providing task-based training can significantly enhance upper limb function in stroke patients (Lohse et al., 2014). Previous research combining VR training with conventional rehabilitation therapy in patients with cerebral infarction has shown long-lasting improvements in upper limb motor function (Laver et al., 2017). In our previous prospective clinical study on rehabilitation intervention of multi-sensory interactive training for patients with cerebral infarction, it was found that virtual reality training combined with conventional rehabilitation therapy could improve upper limb motor function of patients with cerebral infarction and the effect could last for 1 year (Lo et al., 2017a; Zhao et al., 2019c). With the increasing application of VR in stroke rehabilitation, understanding its impact on neuronal activity and motor function becomes crucial.

The primary objective of this study is to investigate the characteristics of oxygenation in the central cortex and upper-limb flexors during grasping tasks in individuals with stroke. fNIRS systems will be used to simultaneously assess blood flow dynamics in the central cortex and upper-limb flexors during grasping tasks, evaluating the heterogeneity of muscle oxygenation activity in the upper-limb flexors and analyzing the activation levels of cerebral cortex functional areas. The secondary objective is to explore the hemodynamic alterations in the cerebral cortex and flexor muscles of the affected upper limb during grasping tasks, induced by the combination of VR and conventional rehabilitation therapy, to attempt to investigate the rehabilitation mechanisms of upper limb motor function in individuals with stroke. Participants with stroke will undergo a combined intervention of VR training and conventional rehabilitation therapy. Changes of hemodynamic in cerebral cortex and upper limb flexor hemodynamics, as well as upper limb motor function, will be evaluated before and after the intervention. Furthermore, correlations between each parameter will be analyzed to gain insights into the relationship between VR training, neuronal activity, and upper limb motor function in stroke patients.

## Methods and design

### Study design

This study follows a longitudinal non-randomized controlled design to examine the synchronous oxygenation characteristics of the central cortex and upper-limb flexors during a grasping task in stroke patients with varying levels of grasp function.

### Study setting

The study is conducted at the Department of Rehabilitation Medicine, First Affiliated Hospital of Sun Yat-sen University in China. Participants are recruited from the inpatient ward, and all interventions are delivered within the Hospital Rehabilitation Department by the hospital staff.

### Recruitment

Eligible patients admitted to the inpatient ward are screened as part of routine assessment. The clinical team identifies suitable participants and provides them with written information about the study. A member of the research team then approaches the potential participants, explains the study further, and obtains written consent from those interested in participating. A screening log is maintained to record details of non-recruited patients and reasons for exclusion.

### Ethics

Data collection will be performed according to the World Medical Association Declaration of Helsinki. The study has received ethical approval from the Human Subjects Ethics Subcommittee of the First Affiliated Hospital of Sun Yat-sen University (Ethical approval number: [2022] 308). Additionally, the study is registered in the Chinese Clinical Trial Registry (Registration No.: ChiCTR2400080619, registered on 2 February 2024). Any significant modifications to the study protocol will be communicated to all relevant parties.

### Participants

Inclusion Criteria:

1. First stroke confirmed by CT or MRI.
2. Medically stable.
3. Age between 40 and 80 years.
4. Onset of stroke occurred between two weeks to three months.
5. Right handedness.
6. No severe cognitive impairment (MMSE ≥ 22 points) and able to follow motor commands.
7. Able to maintain an upright position in an armless seat for at least 30 min.
8. Modified Ashworth grade of the affected upper limb is equal to or less than grade 2.
9. Brunnstrom motor function grade of the affected hand is equal to or greater than grade 3, and able to grasp a 2.5cm3 wood block with effort.
10. Patient and their family members agree to participate in the study and have signed the informed consent.

Exclusion Criteria:

1. Unstable clinical condition.
2. Presence of any neuropsychiatric illness other than stroke.
3. Diagnosis of schizophrenia, bipolar disorder, obsessive-compulsive disorder, personality disorder, or major depression.
4. Presence of skin lesions or infections on the upper limb.
5. History of upper limb fractures, deformities, or finger absence.

Sample Size:

Based on the sample size of previous studies (Huo et al., 2019a), a total of 60 patients following stroke will be enrolled in this study, with 20 patients in each of the three groups.

Group A: Patients who can only grasp and lift a 2.5 cm^3^ wooden block with their hemiplegic hand.
Group B: Patients who can grasp and lift 2.5 cm^3^ and 5 cm^3^ blocks with their hemiplegic hand but cannot lift 10 cm^3^ blocks.
Group C: Patients who can pick up 2.5 cm^3^, 5 cm^3^, and 10 cm^3^ wood blocks with their hemiplegic hand but exhibit abnormalities in speed and coordination during the task.

In addition, a control group of 20 age-matched healthy subjects without neurological diseases or history of upper limb trauma will also be enrolled.

### Experimental procedure

All stroke participants will undergo a comprehensive assessment both before and after the intervention. The assessment will involve the use of fNIRS to simultaneously monitor the blood oxygen parameters of the cerebral cortex and upper limb flexors during a grasping task. Additionally, pre- and post-intervention assessments will include the Action Research Arm Test (ARAT), the upper extremity section of the Fugl-Meyer Assessment (FMA-UE), the Modified Ashworth Scale (MAS), MyotonPRO, and the Modified Barthel Index (MBI).

**FIGURE 1:**
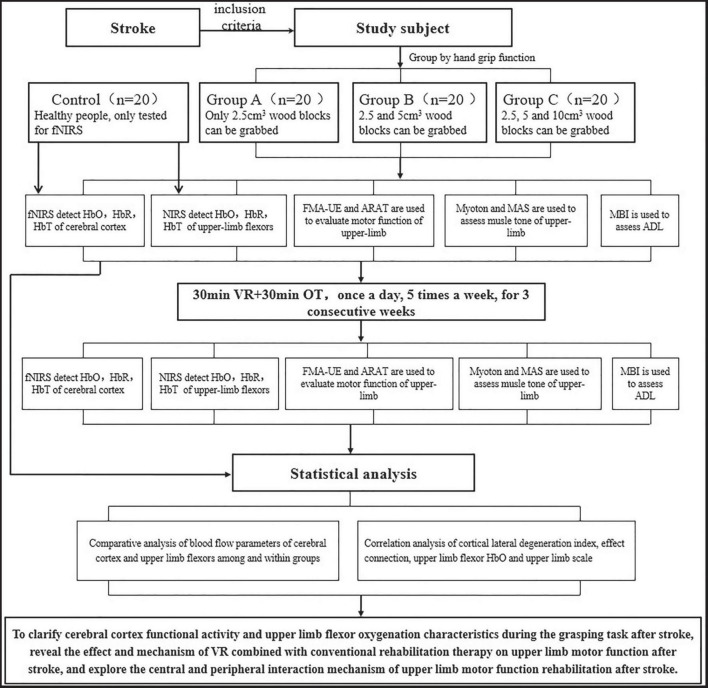
Flow diagram of the study procedure.

During the intervention period, all stroke participants will receive 30 min of VR training and 30 min of occupational therapy once a day, five times a week, for a total of three consecutive weeks. Figure 1 shows the study procedure.

### fNIRS data acquisition and analysis

**FIGURE 2:**
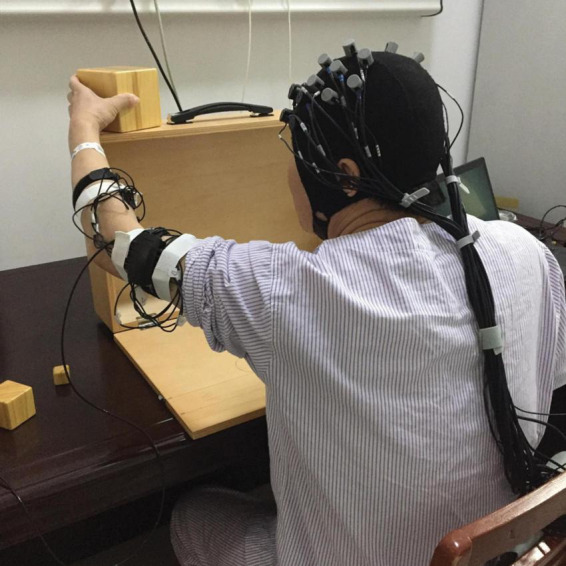
The illustration of fNIRS data acquisition.

**FIGURE 3:**
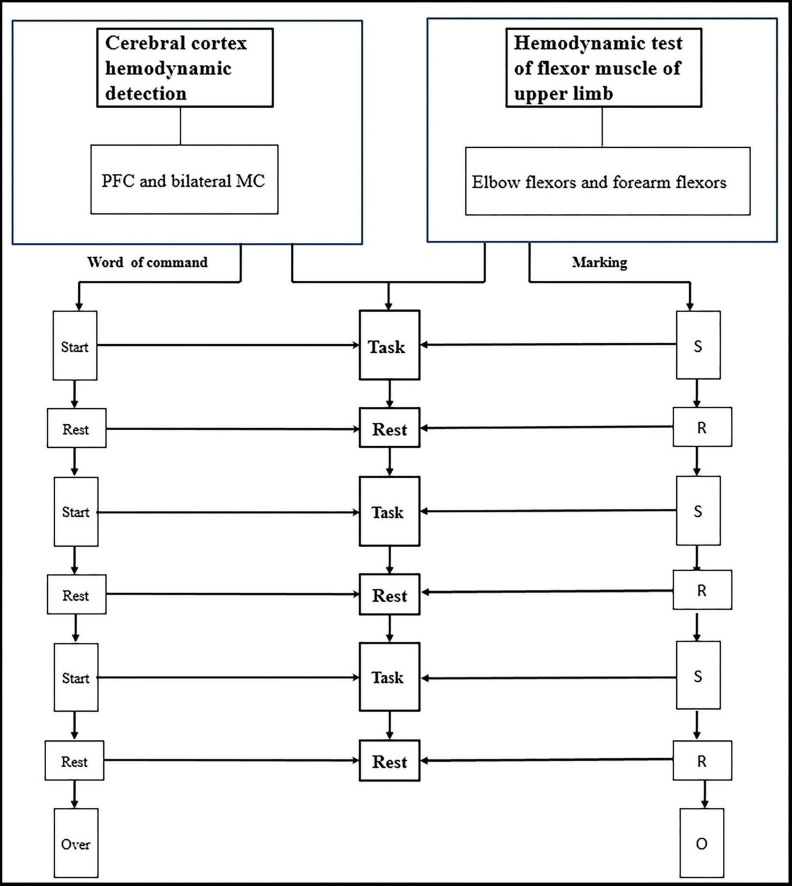
Blood oxygen parameters of cerebral cortex and upper limb flexors are synchronously detected during the grasping task.

The fNIRS data acquisition process is illustrated in Figures 2, 3. The participants were seated comfortably in an adjustable chair within a quiet room. For participants in Group A, they were instructed to grasp a 2.5 cm^3^ wooden block first with their unaffected hand and then with their affected hand. Participants in Group B performed two sets of tests, grasping a 2.5 cm^3^ wooden block and a 5 cm^3^ wooden block, using their unaffected hand first and then their affected hand. Subjects in Group C were asked to sequentially pick up three blocks of different sizes, again using their unaffected hand first and then their affected hand. The control group was required to grasp and lift three different sizes of cubic wooden blocks, first with their right hand and then with their left hand.

Before the actual data collection, a practice session was conducted to familiarize participants with the tasks. The fNIRS data collection followed a block design paradigm. Each condition was preceded by 50 s of rest, which served as a baseline period to stabilize the fNIRS signal. Between each condition, participants were allowed a 2-min break. Each condition consisted of three blocks, with each block consisting of a 20-s motor task period followed by a 20-s rest period. During all rest periods, participants were instructed to keep their focus still on the handle of the ARAT toolbox.

#### fNIRS system and data analysis of cerebral cortex

**FIGURE 4:**
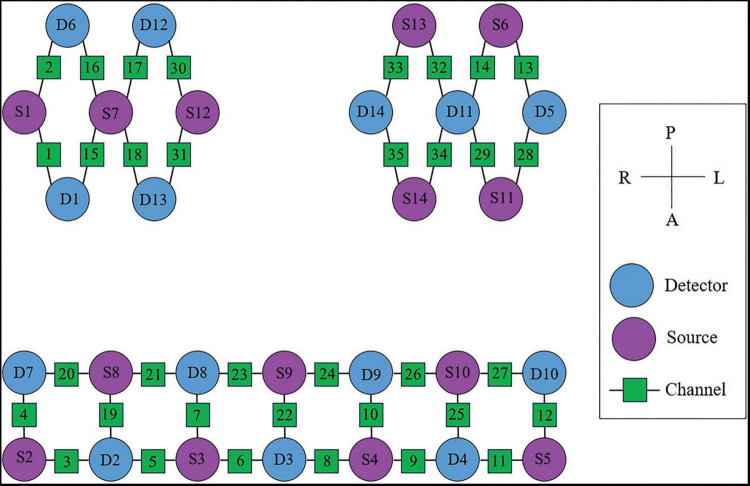
The arrangement of fNIRS channels of cerebral cortex.

In this study, the NirSmart-6000A equipment (Danyang Huichuang Medical equipment Co., Ltd, China) was used to continuously measure and record changes in the concentration of HbO and HbR in the cerebral cortex during the grasping task. The system consisted of near-infrared light sources (LEDs) and avalanche photodiodes (APDs) as detectors. The wavelengths used were 730 nm and 850 nm for the LED and APD, respectively, and the sampling rate was 11 Hz. Fourteen light sources and fourteen detectors were used, forming a total of 35 effective channels (CHs) as shown in Figure 4. The average distance between the light sources and detectors was 3 cm, ranging from 2.7 cm to 3.3 cm. To define the coordinates of the acquired channels, an NIRS-EEG compatible cap from EASYCAP, Herrsching, Germany, based on the international 10/20 system, was used for localization. The brain regions observed in this study were the bilateral prefrontal cortex (LPFC/RPFC), motor cortex (LMC/RMC), and occipital lobe (LOL/ROL), according to existing literature. No detector signal supersaturation occurred during the recording process. The middle point of each channel was defined as the main brain region detected by that channel, and the coordinates of each channel were calibrated using this point as the center of a circle. The Montreal Neurological Institute (MNI) coordinates of each channel were calculated and registered based on the 10-20 system using the NIRSpark (Danyang Huichuang Medical equipment Co., Ltd, China). The corresponding brain regions were then identified in the adult Brodmann Chris Rorden’s MRIcro brain model. The coordinates of each channel and the calibration information of the brain regions are provided in Table 1.

**TABLE 1:** The coordinates of each channel and the calibration information of brain region.

Channel	Sources	Detectors	X	Y	Z	Brodmann Area (Chris rorden’ MRIcro)	Percentage
CH1	S1	D1	52.356	−1.9313	55.131	6−Pre-Motor and Supplementary Motor Cortex	0.9012
CH2	S1	D6	56.299	−23.562	56.601	1−Primary Somatosensory Cortex 3−Primary Somatosensory Cortex	0.4436 0.428
CH3	S2	D2	55.399	41.65	1.9866	45−pars triangularis Broca’s area	0.6744
CH4	S2	D7	59.693	29.199	14.255	45−pars triangularis Broca’s area	0.9135
CH5	S3	D2	41.854	60.848	5.2035	10−Frontopolar area	0.659
CH6	S3	D3	19.831	71.754	8.2877	10−Frontopolar area	0.9521
CH7	S3	D8	29.242	63.37	19.568	10−Frontopolar area	0.7386
CH8	S4	D3	−10.758	73.07	10.168	10−Frontopolar area	1
CH9	S4	D4	−35.079	62.495	11.108	10−Frontopolar area	0.7358
CH10	S4	D9	−21.034	65.834	24.694	10−Frontopolar area	0.8321
CH11	S5	D4	−52.275	42.85	10.496	45−pars triangularis Broca’s area	0.7786
CH12	S5	D10	−56.346	28.713	24.181	45−pars triangularis Broca’s area	0.8309
CH13	S6	D5	−55.973	−23.32	57.463	3−Primary Somatosensory Cortex	0.5484
CH14	S6	D11	−45.237	−23.017	66.994	3−Primary Somatosensory Cortex 4−Primary Motor Cortex	0.4483 0.5211
CH15	S7	D1	42.319	−2.2922	62.568	6−Pre-Motor and Supplementary Motor Cortex	0.8943
CH16	S7	D6	45.142	−23.342	67.067	4−Primary Motor Cortex	0.5904
CH17	S7	D12	33.732	−24.261	73.163	4−Primary Motor Cortex	0.6545
CH18	S7	D13	30.879	−4.5054	68.988	6−Pre-Motor and Supplementary Motor Cortex	1
CH19	S8	D2	46.574	49.425	18.266	46−Dorsolateral prefrontal cortex	0.642
CH20	S8	D7	51.663	35.728	29.39	45−pars triangularis Broca’s area	0.9425
CH21	S8	D8	36.333	51.127	30.619	46−Dorsolateral prefrontal cortex	0.8843
CH22	S9	D3	6.9278	68.503	23.942	10−Frontopolar area	1
CH23	S9	D8	16.238	59.508	34.233	9−Dorsolateral prefrontal cortex	0.6039
CH24	S9	D9	−8.4826	60.969	37.456	9−Dorsolateral prefrontal cortex	0.6577
CH25	S10	D4	−42.728	50.755	24.327	46−Dorsolateral prefrontal cortex	0.6624
CH26	S10	D9	−29.041	53.06	37.191	9−Dorsolateral prefrontal cortex 46−Dorsolateral prefrontal cortex	0.4318 0.5682
CH27	S10	D10	−45.617	35.222	37.851	45−pars triangularis Broca’s area	0.4762
CH28	S11	D5	−50.878	−2.2923	56.073	6−Pre-Motor and Supplementary Motor Cortex	0.9298
CH29	S11	D11	−40.425	−0.87957	63.65	6−Pre-Motor and Supplementary Motor Cortex	0.977
CH30	S12	D12	20.744	−25.16	77.428	4−Primary Motor Cortex	0.6824
CH31	S12	D13	20.445	−2.6141	76.598	6−Pre-Motor and Supplementary Motor Cortex	1
CH32	S13	D11	−34.819	−22.105	73.515	4−Primary Motor Cortex	0.6528
CH33	S13	D14	−23.263	−20.709	77.36	4−Primary Motor Cortex 6−Pre-Motor and Supplementary Motor Cortex	0.4542 0.5458
CH34	S14	D11	−30.534	−1.1513	68.7	6−Pre-Motor and Supplementary Motor Cortex	1
CH35	S14	D14	−19.211	−0.49547	76.226	6−Pre-Motor and Supplementary Motor Cortex	1

Some channels may cover more than one brain region. To save space in this paper, only the brain regions with a coincidence degree higher than 0.4 are listed in this table.

For data analysis, the NirSpark software package, running in MATLAB (The Mathworks, USA), was utilized. The data preprocessing involved several steps, including eliminating irrelevant time intervals, removing non-related artifacts, converting light intensity to optical density, applying a band-pass filter, converting optical density to blood oxygen concentration, and setting parameters for the hemodynamic response function (HRF). The HRF initial time was set to −2 s, and the end time was set to 40 s, with a baseline state from −2 s to 0 s and a single block paradigm from 0 s to 40 s. Both the “start” and “rest” durations were set to 20 s. The blood oxygen concentration data from the three block paradigms were then averaged to generate a block average result. These preprocessing steps allowed for meaningful analysis of the fNIRS data, providing insights into changes in cerebral blood flow and oxygenation during the experimental conditions.

In this study, the Generalized Linear Model (GLM) (Uga et al., 2014) was utilized to analyze the pre-processed time-series data of HbO for each channel and subject. A t-test was performed (P < 0.05) to compare the baseline and task states. The GLM allowed for the establishment of an ideal Hemodynamic Response Function (HRF) specific to each task and subject. The similarity between the experimental and ideal HRF values was evaluated (Chung et al., 2018). The beta value, representing the level of cortical activation for each channel, was used as an estimate of the HRF prediction for the HbO signal (Kawabata et al., 2019). It indicates the peak value of the HRF function and serves as a measure of cortical activation.

Wavelet amplitude (WA) of hemoglobin signal calculated by wavelet transform was used to quantify the intensity of cortical activity. For a single channel, 3 time windows of 20 s were divided to calculate the average WA value in the frequency domain, that is, 3 cortical activity intensity indices could be obtained. The intensity of cortical activity in a cortical region was calculated as the average WA value for all channels in that region. The WA-based laterality metric was calculated by the following equation:


LateralizationIndex=(WAaffected-WAunaffected)/

(WA⁢affected+WA⁢unaffected).

where WA affected denotes the mean WA value in the cortical region of affected hemisphere and WA unaffected denotes the mean WA value in the homologous cortical region of the unaffected hemisphere. The value of WA laterality ranges from 1 to 1. Positive values imply that the affected side cortical activity is greater than the unaffected side, and negative values imply that the unaffected side is greater than the affected side.

#### fNIRS system and data analysis of upper limb flexors

**FIGURE 5:**
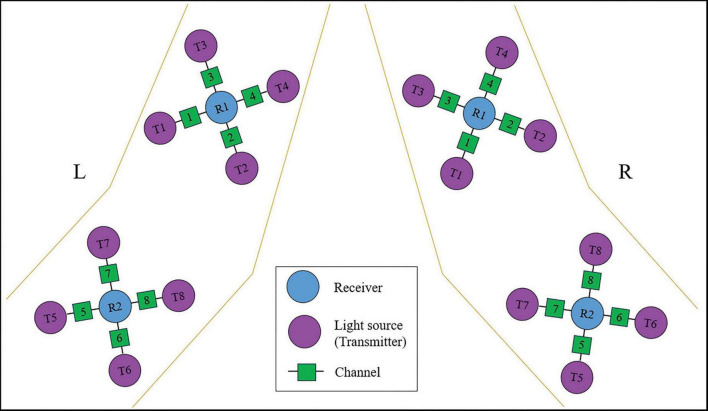
The arrangement of fNIRS channels for upper-limb flexors.

In this study, an OctaMon M system (Artinis, LLC, New York, NY, USA) was used to continuously measure and record changes in the concentration of HbO and HbR in the upper limb flexors during the grasp task. The system consisted of two receivers and eight transmitter probes, forming a total of eight effective channels (CHs) as shown in Figure 5. The distance between the transmitter and receiver probes was 35 mm for the OctaMon M system, and the inter-optode distance was less than 3 mm. The absorption of near-infrared light at two different wavelengths (760.0 nm and 850.0 nm) was measured, and a sampling rate of 10 Hz was used during the measurements. The channel widths were adaptable and ranged from 25 mm to 40 mm. With a 2 × 4 channel configuration, changes in oxyhemoglobin, deoxyhemoglobin, and total hemoglobin values could be regionally monitored.

During the experiments, the OctaMon M system was placed on the flexors of the elbow and wrist to record the parameters of interest. When locating the muscle holder 1 on the flexors of the forearm, the receiver 1 was positioned at the upper one-third distance from the external epicondyle of the humerus to the styloid process of the ulna. When locating the muscle holder 2 on the elbow flexors, the receiver 2 was positioned at the lower one-third distance from the medial epicondyle of the humerus to the acromion. The arrangement of fNIRS channels for the upper limb flexors is shown in Figure 5.

For data analysis, dedicated software called OxySoft from Elst, The Netherlands, was used to analyze the system data. The fNIRS signal was transferred directly to a PC computer using a Bluetooth device. The collected data were exported to a text file and subsequently analyzed using MATLAB software.

### Interventions

In this study, two different rehabilitation interventions were used: Virtual Reality Training (VR) and Conventional Occupational Therapy (OT).

For the VR training, the Arm Guider upper limb rehabilitation training system (Shanghai ZD Medical Technology Co., Ltd, China) was used. This system is specifically designed for individuals with upper limb dysfunction and provides various modes of training, including passive, assisting, active, and resistance training. The goal of VR training is to enhance proprioception, promote brain function remodeling, and reconstruct the neural system network. It combines exercise training with immersive game scenes to improve control, coordination, strength, and joint motion of the upper limbs, as well as train memory, understanding, and spatial structure imagination. During the first training session, the range of movement was individually set up, and the training mode was selected based on the residual motor function of the affected upper extremity. Real-time visual feedback was provided through a monitor located approximately 1 meter away from the patients to assist in task-oriented training. Additionally, real-time auditory feedback was given to indicate the quality of performance. The training sessions were conducted by an experienced occupational therapist for 30 min each, with rest intervals. The VR training was administered once a day, five times a week, for a total of three consecutive weeks.

Conventional OT was also performed by the same occupational therapist who had 15 years of experience in stroke occupational treatment. The OT treatment primarily focused on traditional neuromuscular promotion techniques, including joint compression, muscle extension, upper limb and hand functional training, and activities of daily living (ADL) training. Each OT session lasted for 30 min and was conducted once a day, five times a week, for three consecutive weeks.

### Outcomes

#### Primary outcomes

In this study, two different applications of functional near-infrared spectroscopy (fNIRS) were employed: fNIRS of the cerebral cortex and fNIRS of the upper-limb flexors.

For fNIRS of the cerebral cortex, a Huichuang portable 35-channel fNIRS system was utilized to measure blood oxygen parameters, including HbO, HbR, and HbT. The system used the continuous wavelet transform method to calculate the wavelet amplitude (WA) of signals ranging from 0.01 to 0.08 Hz in each cortical region (Huo et al., 2019a). The phase information was extracted from the signals. The inter-hemispheric lateral degeneration index was then calculated based on WA to evaluate the degree of brain activation transfer from the affected side to the healthy side. Additionally, the extracted phase information was used for effect connection analysis based on dynamic Bayesian inference to assess the strength of the interaction between the cerebral cortex regions (Huo et al., 2019a).

For fNIRS of the upper-limb flexors, a portable muscle oxygen detector called OctaMon from Artinis Medical Systems B. V. was employed. This system utilized an 8-channel near-infrared spectroscopy setup to measure blood oxygen parameters (HbO, HbR, HbT) in the flexor muscles of the upper limb. A distribution map was created based on the measured parameters. The spatial-temporal parameters of blood oxygen were analyzed to evaluate muscle activities.

Overall, both fNIRS applications aimed to investigate blood oxygen parameters in different regions of interest (cerebral cortex and upper-limb flexors) and analyze their characteristics to understand brain activation transfer, cerebral cortex interactions, and muscle activities in the context of the study.

#### Secondary outcomes

The upper extremity section of the Fugl–Meyer assessment (FMA-UE): The FMA-UE has frequently been used to measure UE motor impairment (Prochazka and Kowalczewski, 2015; Qian et al., 2017) and has been reported to yield good intra-rater and inter-rater reliability and construct validity (Page et al., 2012). It is widely used to evaluate motor impairment in the upper extremity and has a Chinese version that is frequently employed in research (Wu et al., 2009).

Action Research Arm Test (ARAT): The ARAT was developed as a performance test to assess upper extremity function and dexterity after stroke (Lyle, 1981). A Chinese version of the ARAT (C-ARAT) has been translated and has been proved to have good validity, reliability (inter-rater reliability = 0.998, intra-rater reliability = 0.987), responsiveness, and predictive ability (Zhao et al., 2019c; Zhao et al., 2019a; Zhao et al., 2019b). The ARAT follows a hierarchical order, where the difficulty of items increases progressively.

MyotonPRO: Muscle tone of biceps and flexor carpi radialis of both upper limbs is evaluated using a portable MyotonPRO instrument. MyotonPRO demonstrated acceptable relative and absolute reliability in a ward setting for patients with acute stroke (Lo et al., 2017b).

Modified Ashworth scale (MAS): The MAS is an assessment tool commonly used to measure spasticity in individuals with stroke. The scale comprises six levels of spasticity severity, including grade 0, 1, 1+, 2, 3, and 4, which is in sequence scored 0, 1, 1.5, 2, 3, and 4 points in this study. This study assessed the flexors of elbow, wrist and fingers. MAS has also been shown to have excellent inter-rater reliability (ICC = 0.686-0.781) and intra-rater reliability (ICC = 0.644-0.748) in assessing spasticity in patients (Meseguer-Henarejos et al., 2018).

Modified Barthel Index (MBI): The MBI is a tool that is commonly used to assess the self-care ability of daily living, which reflects functional capacity, particularly in patients following stroke (Perez et al., 2018). In assessing patients following stroke, the MBI has shown excellent reliability (ICC = 0.88) (Yang et al., 2021).

### Data management

To protect participant confidentiality, all data collected will be assigned unique codes instead of using participants’ names. Data will be recorded on the case report form. The access of all data will be restricted to the principal investigator only. To ensure accuracy in data entry, the principal investigator will meticulously review the data entries for all participants. Monitoring of study conduct and data collection will be performed by a special committee of the University Clinical Trial Unit on a biannual basis. Following the guidelines set by the journal publication policy, the raw data collected during this research study will be securely stored for a period of up to five years after the publication of the initial paper.

### Statistical analysis

Before conducting parametric tests, several steps were taken to prepare and analyze the data. Outliers were identified using normal Q–Q plots and box plots, and data points that were more than three standard deviations from the mean were considered outliers and subsequently excluded from the analysis (Minati et al., 2011). Missing values were addressed using multiple imputation analysis to estimate and replace the missing data points (Blankers et al., 2010).

Descriptive statistics were used to summarize the demographic and clinical characteristics of the study participants. The normality of the data distribution was assessed using the one-sample Kolmogorov-Smirnov test. For comparisons between different groups, chi-square tests were used for categorical variables, while independent t-tests were used for continuous variables.

To evaluate the differences in outcome measures before and after the intervention for participants with stroke, paired sample *t*-tests were employed. The correlations between variables were assessed using either the Pearson correlation coefficient (r) or the Spearman correlation coefficient (ρ), depending on the nature of the data.

The significance of interregional coupling strength between the resting state and different grasping states was evaluated using a one-way analysis of variance (ANOVA). Additionally, χ^2^ tests were utilized to identify statistically significant differences in interregional coupling direction between the resting state and different grasping states.

All statistical analyses were performed using SPSS software (version 20.0; SPSS Inc., Chicago, IL, USA). All tests were two-tailed, and a significance level of *p* < 0.05 was considered statistically significant.

## Discussion

### Summary

The effectiveness and underlying mechanisms of rehabilitation therapy for improving upper limb function of stroke remain uncertain. fNIRS is a validated and reliable neuroimaging technique that can be used as a wearable device during functional activities (Plichta et al., 2006). Previous studies have utilized fNIRS to examine cortical activation and effective connectivity in stroke patients, by different interventions, such as median nerve electrical stimulation (Huo et al., 2019a), limb linkage rehabilitation tasks (Huo et al., 2019b) and musical finger movement rehabilitation gloves (Li et al., 2020). However, there is a lack of research on the neurophysiological activity of the cerebral cortex function region, as detected by fNIRS, during grasping tasks in stroke patients. Based on previous findings from EEG studies, which suggest that stroke patients have higher cognitive demands during grasping tasks compared to healthy individuals (Chen et al., 2018), it is hypothesized that the activation patterns and intensity of cerebral cortex regions in stroke patients during grasping tasks changed. Additionally, considering the weakened muscle strength and coordination disorders typically observed in the affected side of stroke patients, it is hypothesized that the hemodynamics of the upper-limb flexors undergo changes, including increased oxygen consumption, during grasping tasks after a stroke.

This study aimed to investigate the synchronous oxygenation characteristics of the central cortex and upper-limb flexors during different grasping tasks in individuals who have experienced a stroke. In addition, the study aimed to explore the hemodynamic changes in the cerebral cortex and flexor muscles of the affected upper limb during grasping tasks, brought by the combination of VR and conventional rehabilitation therapy, to try to investigate the rehabilitation mechanisms of upper limb motor function in individuals with stroke.

### Limitations

There were several important limitations in this study that warrant consideration. Firstly, the trial sample size was relatively small. A small sample size can impact the statistical power of the study and may limit the ability to draw robust and generalizable conclusions from the findings. Secondly, the study design employed a non-randomized clinical trial approach, and participants were recruited using convenience sampling from the inpatient population with stroke. This could limit the generalizability of the findings. Thirdly, the study primarily focused on the assessment of hemodynamic changes during grasping motor tasks. Consequently, the understanding of the intervention’s effects on overall motor function may be limited by this narrow focus on specific upper limb tasks. Lastly, the two functional near infrared spectroscopy detection systems was at different sampling rate during the measurements.

### Strengths and relevance

This study has several strengths. Firstly, it aims to investigate the synchronous oxygenation characteristics of both the central cortex and upper-limb flexors during a grasping task in individuals with stroke. The findings from this research have the potential to significantly contribute to our understanding of motor function and recovery in individuals with stroke. It may uncover valuable insights into the underlying mechanisms of motor control and rehabilitation. Secondly, the utilization of two functional near infrared spectroscopy detection systems enables simultaneous monitoring of blood oxygen parameters in both regions, providing a comprehensive assessment of oxygenation dynamics. Thirdly, the study employs a combination of quantitative and qualitative approaches to assess the outcomes. This comprehensive evaluation enhances the interpretation of the findings, capturing both the quantitative metrics and the qualitative aspects of synchronous oxygenation characteristics.
